# Spatial Co-Morbidity of Childhood Acute Respiratory Infection, Diarrhoea and Stunting in Nigeria

**DOI:** 10.3390/ijerph19031838

**Published:** 2022-02-06

**Authors:** Olamide Seyi Orunmoluyi, Ezra Gayawan, Samuel Manda

**Affiliations:** 1 Department of Statistics, Federal University of Technology, Akure 340271, Nigeria; seyiolamide8@gmail.com (O.S.O.); egayawan@futa.edu.ng (E.G.); 2 Department of Statistics, University of Pretoria, Pretoria 0028, South Africa; 3 Biostatistics Research Unit, South African Medical Research Council, Pretoria 0001, South Africa

**Keywords:** shared component, Bayesian analysis, acute respiratory infection, Nigeria, diarrhoea

## Abstract

In low- and middle-income countries, children aged below 5 years frequently suffer from disease co-occurrence. This study assessed whether the co-occurrence of acute respiratory infection (ARI), diarrhoea and stunting observed at the child level could also be reflected ecologically. We considered disease data on 69,579 children (0–59 months) from the 2008, 2013, and 2018 Nigeria Demographic and Health Surveys using a hierarchical Bayesian spatial shared component model to separate the state-specific risk of each disease into an underlying disease-overall spatial pattern, common to the three diseases and a disease-specific spatial pattern. We found that ARI and stunting were more concentrated in the north-eastern and southern parts of the country, while diarrhoea was much higher in the northern parts. The disease-general spatial component was greater in the north-eastern and southern parts of the country. Identifying and reducing common risk factors to the three conditions could result in improved child health, particularly in the northeast and south of Nigeria.

## 1. Introduction

Children in sub-Saharan Africa suffer from the simultaneous occurrence of multiple diseases. Disease co-occurrence is partly attributed to common risk factors such as poor sanitation and water quality, air pollution, and poor access to breast milk and nutrient-dense foods [1,2,3,4]. Several studies have shown that growth faltering is related to diarrheal morbidity in children under 5 years of age in low-income settings [5,6,7,8]. While evidence of associations between child growth retardation and acute respiratory infection is not conclusive [5,7], several studies have found a significant association between diarrhoea and the risk of ARI in children [3,9,10]. The association is more pronounced in malnourished children as they are more susceptible to diarrhoea, and subsequent dehydration and micronutrient loss [3,11]. The combined effect weakens the immune system, further predisposing the child to more infections. Malnourishment in children leads to growth faltering (Stewart et al., 2013). An ecological analysis of the spatial co-occurrence of ART, diarrhoea, and stunting is important to public health policymakers, in that identifying and reducing common risk factors to these three conditions could result in a very significant improvement in child health.

Previous ecological studies on child illnesses in sub-Saharan Africa have mostly used univariate spatial statistics methods, even if multiple diseases were analysed [1,12,13,14,15]. A few studies have been conducted to model multiple diseases in children using joint spatial models [16,17,18,19]. Joint spatial models are of both methodological and epidemiological importance, in that they avoid the specific problems of multiple testing and the identifiability issues in random effect parameter estimations [20,21,22]. A widely applied spatial joint model is the shared-component model [21], which allows for separation of the area-specific risks of each disease into the underlying spatial pattern, which is common to all diseases, and another, which is a disease-specific component to capture the difference of disease-spatial pattern from the spatial shared component. This spatial joint model was used here to assess the state-level co-occurrence of ARI, diarrhoea, and stunting among children aged under 5 years. A total of 61,579 children from the 2008, 2013, and 2018 Nigeria Demographic and Health Surveys (DHSs) were used. We analysed each of the three survey datasets, as well as the pooled data, using Bayesian inference employing the integrated nested Laplace approximation approach (INLA) [23]. To the best of our knowledge, no other study has examined the trends over time in spatial variations of some of the common diseases among children under the ages of five years in sub-Saharan Africa. 

## 2. Methods

### 2.1. Data Source

The study used data from the 2008, 2013, and 2018 Nigeria Demographic and Health Surveys (NDHSs). The NDHSs are nationally representative household surveys utilizing a multistage stratified cluster sampling methodology. They provide data for a wide range of indicators in the areas of population, health, and nutrition. Usually, they involve a sample of women aged 15 to 49 years in both urban and rural households drawn from all 36 states and the Federal Capital Territory (FCT) of Nigeria. The 36 states and the FCT were used as the spatial layer. Information on an episode of illnesses in the two weeks preceding the survey implementation on all alive children aged 0–59 months was also collected. The variabales that were considered in this study are described in Table 1.

### 2.2. Statistical Method

Suppose $Y_{ijd}$ is the status of disease d on child j in state $i\;\left(i=1,\ldots ,37;\;j=1,\ldots ,\;n_{i};\;d=1\;\left(ARI\right),\;2\left(diarrhoea\right),\;3\left(stunting\right)\right)$ and we assign it a value of 1 if the child had the disease and 0 otherwise where $n_{i}$ is the number of children that were sampled in state i. Furthermore, suppose $X_{ijd}$ is a vector of covariates associated with child  ij for disease d. The covariates $X_{ij}$ are used to explain variations in disease risk through the associated disease-specific fixed-effects parameters $\;\beta_{d}$. We also account for the unobserved and underlying disease risk through a state random effect, $U_{id}$. The disease status outcome $Y_{ijd}$ is treated as a Bernoulli random variable with parameter $\;\pi_{ijd}$ being the probability of child ij having disease  d. The effect of the observed covariates $X_{ijd}$ and unobserved effects in $U_{id}$ are modelled using a logit function as follows:

(1) $$log\left(\frac{\pi_{ijd}}{1-\pi_{ijd}}\right)=\alpha_{d}+\beta \prime_{d}X_{ijd}+U_{jd}$$

where $\alpha_{d}$ and $\beta_{d}$ are the disease-specific baseline and fixed effect risks associated with the covariate vector $\;X_{ijd}$, respectively. The state random effect $U_{jd}$ may exhibit spatial dependence and disregarding it in the modelling and estimation procedures could lead to biased and inefficient inference. Thus the state random effect is spatially structured.

To investigate state-level co-morbidity among the three childhood illnesses, we use the spatial shared component model proposed by Knorr-Held and Best [27]. The model allows for the splitting of the state random effect on a disease d into an underlying disease-general burden common to all the three diseases and a disease-specific component that could be considered as the deviation from the common state pattern. To also assess pair-wise disease co-occurrence at the state level, we added three state shared random effects, namely ARI and diarrhoea, ARI and stunting, and diarrehoea and stunting. Thus, the final model for the state-level co-occurrence of ARI and diarrhoea, ARI and stunting is as follows:

(2) $$logit\left(\pi_{ij1}\right)=\alpha_{1}+X_{ij}^{⊤}\beta_{1}+\vartheta \upsilon_{i\left(12\right)}+\theta \upsilon_{i\left(13\right)}+\partial_{1}\upsilon_{i}+s_{i1}$$

(3) logitπij2=α2+Xij⊤β2+1ϑυi12+δυi23+∂2υi+si2

(4) logitπij3=α3+Xij⊤β3+1θυi13+1δυi23)+∂3υi+si3

where now the state random effect $U_{id}$ in (1) is decomposed into an underlying disease-general state common pattern and disease-specific component. For example, in (2) which is the model for ARI, there are three spatial shared components as foloows; $\vartheta \upsilon_{i\left(12\right)}$ (ARI and diarrhoea), $\theta \upsilon_{i\left(13\right)}$ (ARI and stunting) and $\partial_{1}\upsilon_{i}$ (ARI, diarrhoea and stunting) and an ARI-specific state-level effect $s_{i1}$ Similarly for diarrhoea and stunting Equations (3) and (4), respectively. Parameters ϑ, θ,δ and ∂ are gradient weights that allow each disease to have a unique association with the underlying disease state pattern. The spatial shared components are proxies for one or more common risk factors such as air pollution, poor sanitation and hygiene, unclean and unsafe drinking water that contribute to child diarrhoeal diseases and ill health.

Model estimation was done with the Bayesian paradigm under the intrinsic conditional autoregressive (ICAR) normal model for the state-level random effects. The parameters of the spatial shared-component model in (2) were estimated using integrated nested Laplace approximation [23]. We fitted the models using R-INLA in R statistical software [27]. Prior specifications followed the ideas in the previous applications of the model as in [16,17,18,19,20,21,22]. The model assessment was based on deviance information criterion (DIC) [28]. 

## 3. Results

Table 2 shows the distribution of the characteristics of the children studied. A total of 61,579 children under 5 years of age were included, of which 23,851, 27,524, and 10, 204 were from 2008, 2013, and 2018, respectively. Out of the 61,579 children considered, 2923 had ARI, 6675 had diarrhoea, and 8940 were stunted. A total of 1013 had ARI and diarrhoea, 468 had ARI and stunting, 1054 had diarrhoea and were stunted, while 173 suffered from all of the illnesses. A larger percentage (68%) of children was from a rural settlement, while 46% of their mothers had no education.

**Figure 1:**
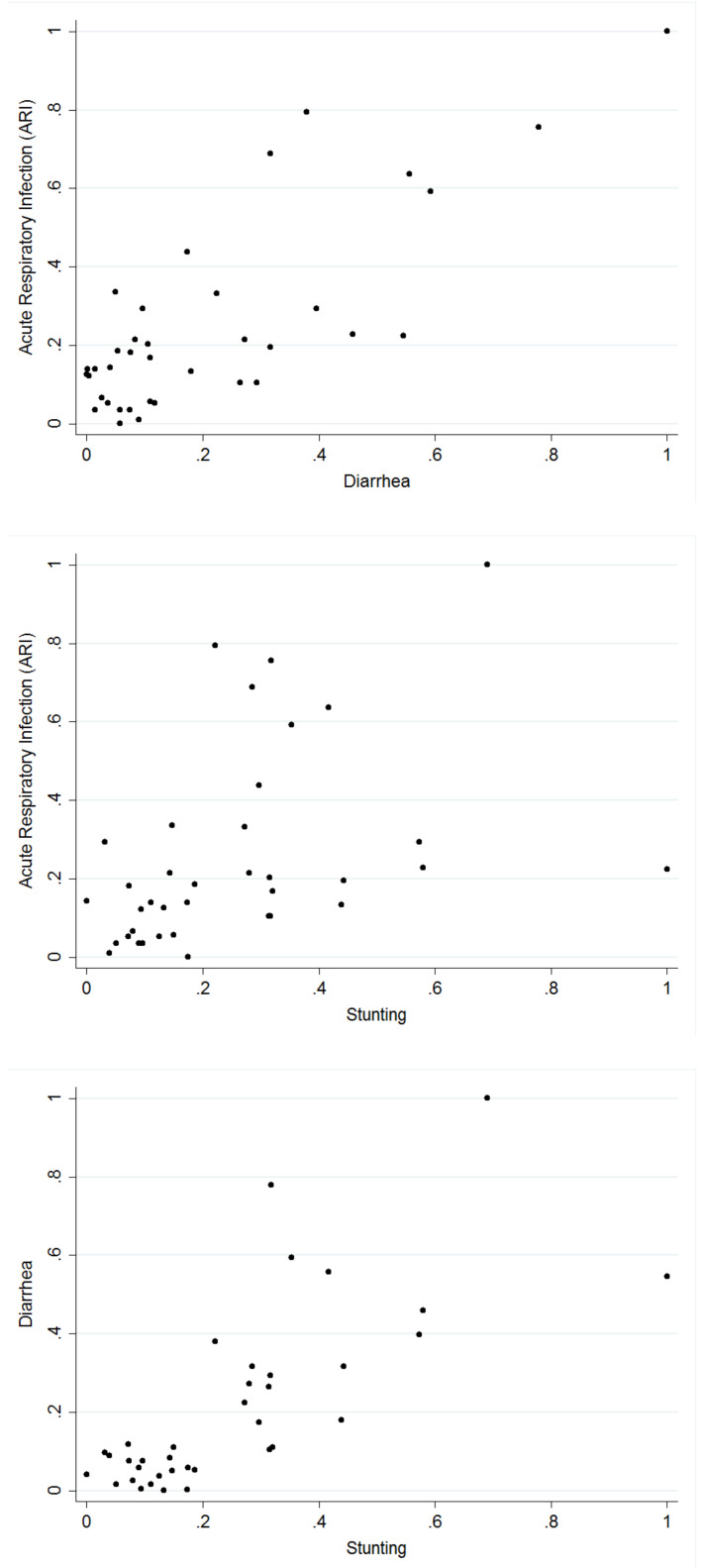
Pairwise scatter plots between the proportions of respiratory infection (ARI), diarrhoea, and stunting based on the 37 locations.

Table 3 presents the tetrachoric correlation coefficients for each pair of the three health indicators computed. The tetrachoric correlation measures were in agreement with the binary outcomes. A strong correlation exists between ARI and diarrhoea. In the case of ARI and stunting, and diarrhoea and stunting, the correlation at the national level was weak, but when estimated for each state (estimates not presented), the results showed significant relationships in some of the states. Thus, correlation estimates at the national level concealed information on possible local variations. Figure 1 shows, for each pair of the diseases, the empirical correlation for the proportion of children who suffered from the diseases in the different states of Nigeria.

Table 4, Table 5 and Table 6 present the odds ratios for the fixed effects for each of the diseases from the surveys carried out in 2008, 2013, and 2018, respectively. Children from urban settlements in 2013 were less likely to be stunted. In 2018, children from urban settlements were more likely to have diarrhoea. Children whose mothers attained at least a primary level of education were less likely to have any of the diseases in all the survey years. This was also true for children from rich households and female children. Estimates also show that children between the age of 12 to 23 months were more likely to have diarrhoea in all the survey years, and as the mothers’ age increased, the likelihood of any of the diseases decreased.

Table 7 presents the odds ratio for the fixed linear effects for each of the diseases considered, including the 95% credible intervals using the combined dataset. The findings show that children who were between 12 to 23 months were less likely to suffer from ARI and stunting, but more likely to suffer from diarrhoea, while children who were 24 months or older had a significantly lower likelihood of suffering from any of the diseases. Children whose mothers attained at least a primary level of education were less likely to have suffered from the diseases, although the estimate was not significant for ARI. Surprisingly, children from an urban settlement were more likely to have diarrhoea when compared with children from a rural settlement. Estimates for ARI and stunting based on settlement (urban or rural) were not significant. However, children from poorer households and above were less like to have any of the diseases, and the estimates were significant. Female children had a lower likelihood of having any of the diseases. As mothers’ age increased, the children were less likely to contract any of the illnesses. In addition, the results show that compared with 2008, the odds for any of the diseases were significantly lower in 2013, but for 2018, the estimates were only lower for ARI and diarrhoea. 

**Figure 2:**
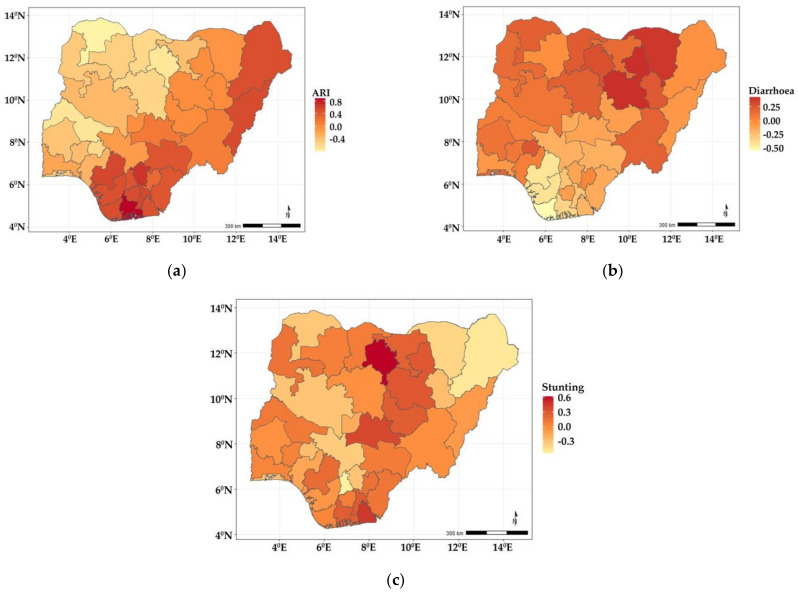
Maps showing posterior estimates of disease-specific spatial effects: (**a**) ARI; (**b**) diarrhoea; (**c**) stunting.

The findings from the spatial effects for the specific diseases as estimated from the shared component model are presented in Figure 2. The maps reveal that whereas diarrhoea has higher odds of occurring among children living in the northern states, ARI is more widespread in the southern fringe. Specifically, children residing in Benue, Enugu, Anambra, Ebonyi, Imo, Abia, Cross River, Akwa Ibom, Rivers, Bayelsa, Delta, Edo, and two states from the northern fringe (Borno and Adamawa) were more likely to have suffered more from ARI, but were less likely in the states belonging to north and southwestern regions of the country. In the case of diarrhoea (Figure 2b), the odds were higher among children living in Yobe, Gombe, and Ekiti, but less likely for those in Edo, Delta, and Bayelsa, while for stunting (Figure 2c), the odds were higher in some neighbouring northern states—Kano, Plateau, Jigawa, Bauchi, Nasarawa, and two southern states (Akwa Ibom and Edo).

**Figure 3:**
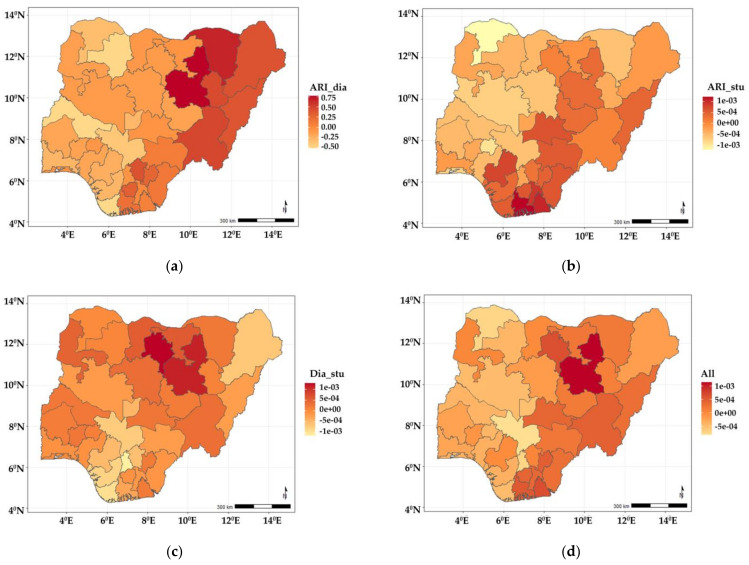
Maps showing posterior estimates of spatial shared effects: (**a**) ARI and diarrhoea; (**b**) ARI and stunting; (**c**) diarrhoea and stunting; (**d**) all three illnesses.

Figure 3a–d show the estimated spatial shared components across the states in Nigeria. The findings show that the shared effects of ARI and diarrhoea (Figure 3a) were higher in neighbouring north-eastern states of Yobe, Bauchi, Borno, Gombe, Adamawa, Taraba, and a few neighbouring southern states (Enugu, Ebonyi, and Anambra) but lower among children residing in states belonging to the northwest and south-west regions of the country. The shared effects for ARI and stunting (Figure 3b) showed higher odds among children living in Adamawa, Bauchi, Plateau, Nasarawa, Benue, Cross-River, Enugu, Ebony, Abia, Imo, Rivers, Edo, Delta, and Bayelsa, but were lower among those residing in the north-western and south-western regions of the country. As for diarrhoea and stunting (Figure 3c), the shared effects revealed higher odds among children living in Bauchi, Kano, and some of the neighbouring states, while they were less likely in Borno, Kogi, Enugu, Abia, Anambra, Imo, Edo, Delta, and Bayelsa. The shared effects for the three diseases presented in Figure 3d revealed higher odds of suffering from the three diseases among children living in Bauchi, Kano, Taraba, Nasarawa, Adamawa, and some south southern states (Enugu, Ebonyi, Imo, Rivers, Abia, and Akwa Ibom). Year-specific disease estimates of disease-specific and shared state components are shown in Appendix A, and similar patterns are observed.

## 4. Discussion

This study simultaneously examined the common and disease-specific spatial components of ARI, diarrhoea, and stunting in children under the age of 5 years old in Nigeria. We employed the shared spatial component model, which allowed us to split the geographical risk of child illnesses into common and uncommon spatial patterns. Childhood illness data obtained from 2008, 2013, and 2018 Nigerian Demographic and Health Surveys were used in the analyses. Our analysis could be considered an extension to the univariate spatial statistical application on childhood illnesses in Nigeria [19,29,30,31], most of which have relied on using univariate spatial models [12,34].

Our study found that the state-level co-morbidity of ARI and diarrhoea; ARI and stunting; diarrhoea and stunting; and ARI, diarrhoea, and stunting were predominant in the northeast and southern parts of the country. Our findings are consistent with previous works that found a higher prevalence of stunting and diarrhoeas among children living in the northern part of the country [33,35,36]. Our findings of higher ARI-related co-morbidity in the southern parts of the country could be linked to oil spillage, leading to adverse air pollution and sand, which could adversely impact child health [32,33,34,35,36,37,38,39]. Regarding the effect of covariates, our findings are consistent with previous studies [40,41,42,43].

This study has some limitations, including that the child illnesses analysed were based on self-reporting from mothers and caregivers. Inaccuracies due to errors in reporting and recall could have been introduced into the data [44]. A longer recall period of infections may underestimate their prevalence rates, as well as missing disease data [45], which could adversely affect the estimate of the health indicators. We did not undertake any quality assessment of the data not accounting for the missing data. However, as the NDHS dataset analyses here ascertained acute respiratory infection and diarrhoea outcomes using mothers or caregiver-reported symptoms for a 2-week recall period, we believe the observed spatial patterns would not be affected.

## 5. Conclusions

Understanding the geographical pattern of the co-occurrences of childhood illnesses at the sub-national level could support local governments in formulating policies and interventions for child health. Using the spatial shared component modelling approach, this study jointly analysed acute respiratory infection, diarrhoea, and stunting in Nigeria. The state-specific risks of each disease was decopmposed into an underlying disease-common state component, which is common to all the three diseases and a disease-specific component, which captures the disease-specific spatial pattern that deviates from the shared spatial pattern. In this way, our findings could support local-level policymakers in devising interventions according to the patterns of child illnesses, whether by integrated approaches or specialized single disease initiatives.

## Figures and Tables

**Table 1:** Variables used for this study.

Variables	Definition
Place of residence	Type of place of residence where the household resides, either urban or rural.
Mother’s level of education	Highest level of education the mother received. This is a standardized variable providing level of education in the following categories: no education, primary, secondary, or higher.
Wealth index	The Wealth Index is a composite measure of the cumulative living standard of a household. It is calculated using data on a household’s ownership of a selected set of assets, such as televisions, bicycles, and cars; dwelling characteristics such as flooring material; type of drinking water source; and toilet and sanitation facilities. The Wealth Index considers characteristics that are related to wealth status, avoiding variables that do not represent an asset, or outcome variables. They are combined using principal components analysis (PCA) methods, and the first component is used as the household Wealth Index [24].
Child’s gender	Sex of the child.
Child’s age	Child’s age in months.
Mother’s age	Mother’s age in years.
Diarrheoa	Whether the child had diarrhoea in the last 24 h or within the last two weeks before the survey.
Stunting	The anthropometric index for height-for-age used to determine the nutritional status of the child is based on Z-scores computed according to the WHO [25] standard [26]. A child whose Z-score is below −2 standard deviation (−2SD) of the reference population is said to be stunted.
Acute respiratory infection	Whether the child suffered from short, rapid breathing, which was chest-related, and/or difficult breathing, which was chest-related in the last weeks before the survey.

**Table 2:** Characteristics of the study population.

Response Variables	Year		
	2008	2013	2018
Total Number of Children	23,851	27,524	10,204
*Disease*			
ARI	1134	1114	675
Diarrhoea	2495	2868	1312
Stunting	4055	3453	1432
ARI and diarrhoea	371	448	194
ARI and stunting	459	393	202
Diarrhoea and stunting	205	166	97
All illnesses	80	64	29
*Residence*			
Rural	17,298	18,206	6633
Urban	6553	9318	3571
*Mother’s education*			
No education	11,714	12,661	4234
Primary	5471	5594	1771
Secondary or tertiary	6666	9269	4199
*Wealth Index*			
Poorest	6193	6020	2283
Poor	10,252	11,813	4570
Rich	7406	9691	3351
***Child gender***			
Male	12,040	13,854	5123
Female	11,811	13,670	5081
*Child’s age (months)*			
Less than 1	6357	8762	2533
12 to 23	4629	6916	2054
24 to 59	12,865	15,085	5617

**Table 3:** Tetrachoric correlation among acute respiratory infection, diarrhoea, and stunting.

	Correlation	Standard Error	*p*-Value
Diarrhoea/ARI	0.371	0.012	0.0000
Diarrhoea/Stunting	−0.024	0.011	0.0274
ARI/Stuntin	−0.023	0.014	0.0973

**Table 4:** The odds ratio estimates of the covariate effects and their 95% credible interval for 2008.

Variables	ARI		Diarrhea		Stunting	
	OR	95% CI	OR	95% CI	OR	95% CI
*Residence*						
Urban	1.032	0.878, 1.214	1.039	0.921, 1.174	1.028	0.931, 1.135
*Mother’s Education*						
No education	1	1	1	1	1	1
Primary	0.803	0.676, 0.951	0.820	0.726, 0.925	0.888	0.805, 0.979
Secondary or tertiary	0.594	0.486, 0.723	0.621	0.535, 0.721	0.593	0.528, 0.666
*Wealth Index*						
Poorest	1	1	1	1	1	1
Poorer or middle	0.782	0.681, 0.899	0.740	0.673, 0.815	0.822	0.758, 0.892
Richer or richest	0.692	0.559, 0.855	0.590	0.504, 0.692	0.635	0.559, 0.720
*Female child*	0.785	0.699, 0.881	0.708	0.652, 0.770	0.896	0.837, 0.959
*Child’s age (months)*						
less than 12	1	1	1	1	1	1
12 to 23	0.964	0.827, 1.121	1.294	1.161, 1.440	0.925	0.839, 1.020
24 to 59	0.534	0.469, 0.607	0.545	0.496, 0.599	0.841	0.780, 0.908
*Mother’s age*	0.915	0.911, 0.921	0.944	0.940, 0.948	0.959	0.955, 0.962

**Table 5:** The odds ratio estimates of covariate effects and their 95% Credible Interval for 2013.

Variables	ARI		Diarrhea		Stunting	
	OR	95% CI	OR	95% CI	OR	95% CI
*Residence*						
Urban	1.061	0.889, 1.265	1.097	0.975, 1.233	0.889	0.797, 0.990
*Mother’s Education*						
No education	1	1	1	1	1	1
Primary	0.941	0.789, 1.121	0.854	0.759, 0.96	0.964	0.866, 1.072
Secondary or tertiary	0.744	0.615, 0.9	0.617	0.542, 0.703	0.692	0.614, 0.781
*Wealth Index*						
Poorest	1	1	1	1	1	1
Poorer or middle	0.652	0.563, 0.755	0.741	0.673, 0.816	0.825	0.755, 0.902
Richer or richest	0.448	0.356, 0.562	0.694	0.595, 0.809	0.647	0.561, 0.744
*Female child*	0.746	0.664, 0.838	0.845	0.782, 0.913	0.912	0.85, 0.979
*Child’s Age (months)*						
Less than 12	1	1	1	1	1	1
12 to 23	0.940	0.808, 1.092	1.278	1.155, 1.414	0.877	0.791, 0.971
24 to 59	0.482	0.422, 0.55	0.517	0.473, 0.566	0.773	0.713, 0.838
*Mother’s Age*	0.914	0.909, 0.92	0.944	0.94, 0.947	0.948	0.944, 0.951

**Table 6:** The odds ratio estimates of covariate effects and their 95% Credible Interval for 2018.

Variables	ARI		Diarrhea		Stunting	
	OR	95% CI	OR	95% CI	OR	95% CI
*Residence*						
Urban	1.050	0.84, 1.309	1.234	1.045, 1.455	0.826	0.709, 0.960
*Mother’s Education*						
No education	1	1	1	1	1	1
Primary	0.956	0.750, 1.216	0.785	0.652, 0.943	0.649	0.544, 0.773
Secondary or tertiary	0.685	0.536, 0.873	0.648	0.540, 0.778	0.522	0.439, 0.619
*Wealth Index*						
Poorest	1	1	1	1	1	1
Poorer or middle	0.536	0.439, 0.653	0.732	0.633, 0.846	0.977	0.848, 1.125
Richer or richest	0.528	0.397, 0.702	0.555	0.445, 0.692	0.621	0.505, 0.762
*Female child*	0.794	0.679, 0.927	0.883	0.785, 0.993	0.922	0.825, 1.029
*Child’s Age (months)*						
Less than 12	1	1	1	1	1	1
12 to 23	0.887	0.719, 1.092	1.220	1.044, 1.425	0.856	0.729, 1.005
24 to 59	0.538	0.451, 0.642	0.523	0.456, 0.599	0.797	0.703, 0.904
*Mother’s Age*	0.934	0.926, 0.941	0.951	0.946, 0.957	0.960	0.955, 0.965

**Table 7:** The odds ratio estimates of the covariate effects and their 95% credible interval for combined data.

Variables	ARI		Diarrhoea		Stunting	
	OR	95% CI	OR	95% CI	OR	95% CI
Residence						
Urban	1.037	0.937, 1.148	1.109	1.0304, 1.193	0.94	0.880, 1.003
*Mother’s education*						
No Education	1	1	1	1	1	1
Primary	0.899	0.807, 1.000	0.83	0.769, 0.896	0.891	0.834, 0.952
Secondary or Tertiary	0.702	0.624, 0.789	0.656	0.602, 0.714	0.646	0.599, 0.696
*Wealth Index*						
Poorest	1	1	1	1	1	1
Poorer or Middle	0.727	0.665, 0.793	0.764	0.719, 0.811	0.883	0.835, 0.933
Richer or Richest	0.576	0.504, 0.659	0.635	0.576, 0.670	0.667	0.612, 0.726
*Female child*	0.791	0.736, 0.850	0.807	0.767, 0.849	0.921	0.881, 0.963
*Child’s age (months)*						
less than 12	1	1	1	1	1	1
12 to 23	0.958	0.871, 1.053	1.28	1.198, 1.367	0.922	0.864, 0.984
24 to 59	0.527	0.485, 0.571	0.538	0.507, 0.570	0.832	0.791, 0.876
*Mother’s age*	0.923	0.920, 0.926	0.947	0.945, 0.949	0.959	0.957, 0.9615

**Figure A1:**
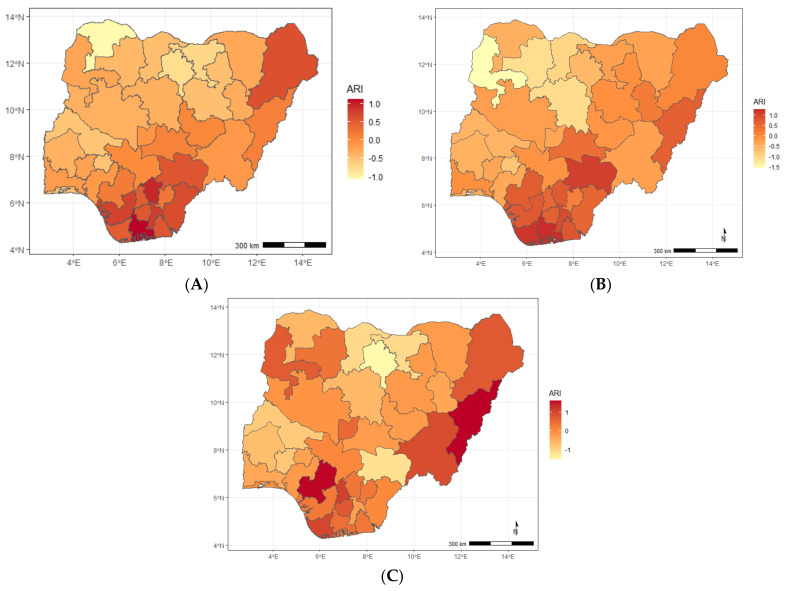
Maps showing posterior estimates of ARI-specific spatial effect by year of NDHS implementation; (**A**) 2008, (**B**) 2013, (**C**) 2018.

**Figure A2:**
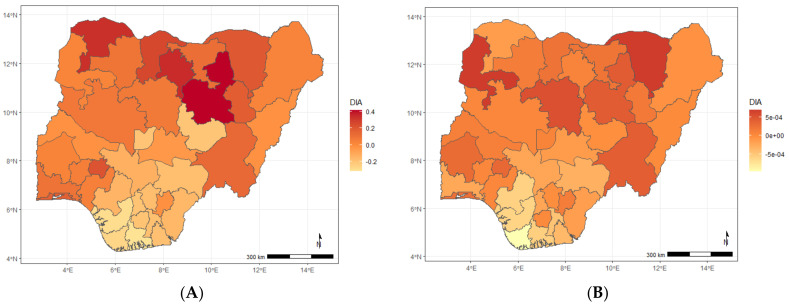

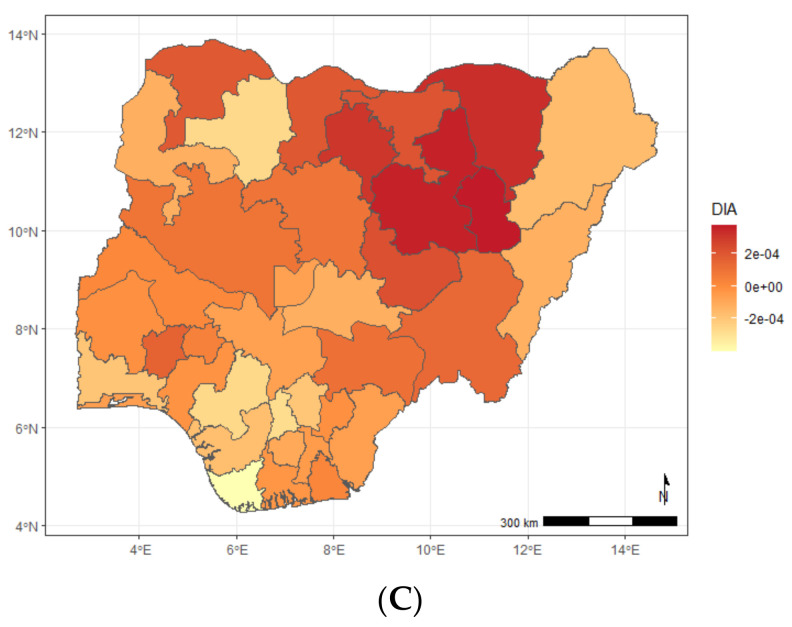
Maps showing posterior estimates of diarrhoea-specific spatial effect by year of NDHS implementation; (**A**) 2008, (**B**) 2013, (**C**) 2018.

**Figure A3:**
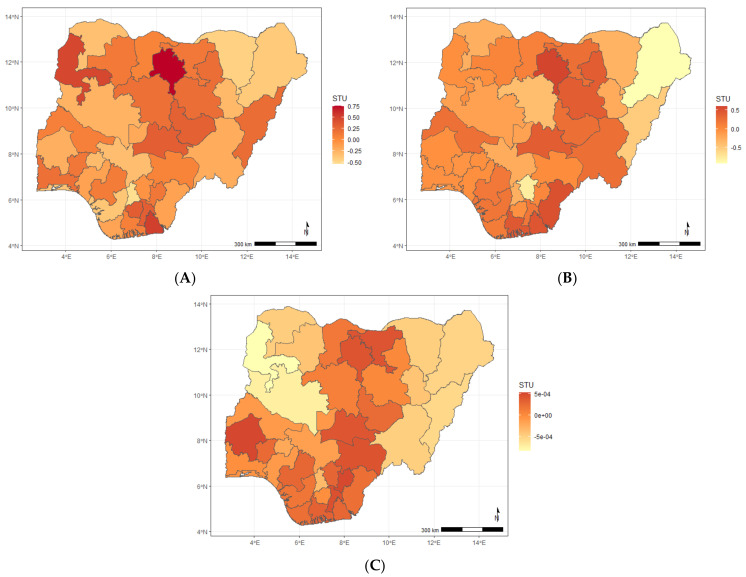
Maps showing posterior estimates of stunting-specific spatial effect by year of NDHS implementation; (**A**) 2008, (**B**) 2013, (**C**) 2018.

**Figure A4:**
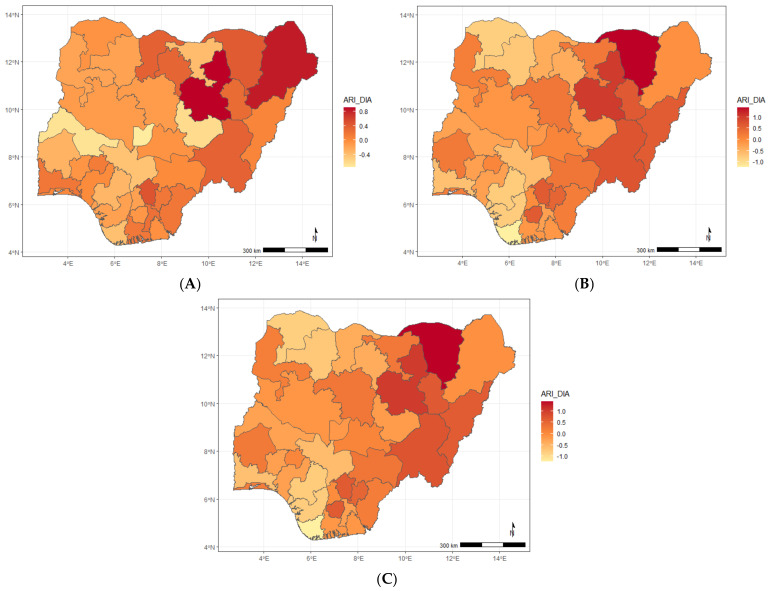
Maps showing posterior estimates of the ARI and diarrhoea spatial shared effect by year of NDHS implementation; (**A**) 2008, (**B**) 2013, (**C**) 2018.

**Figure A5:**
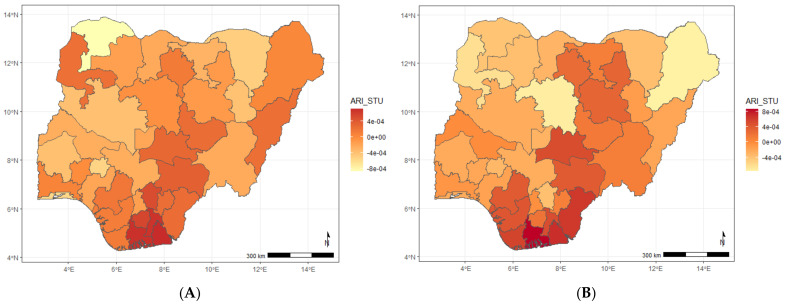

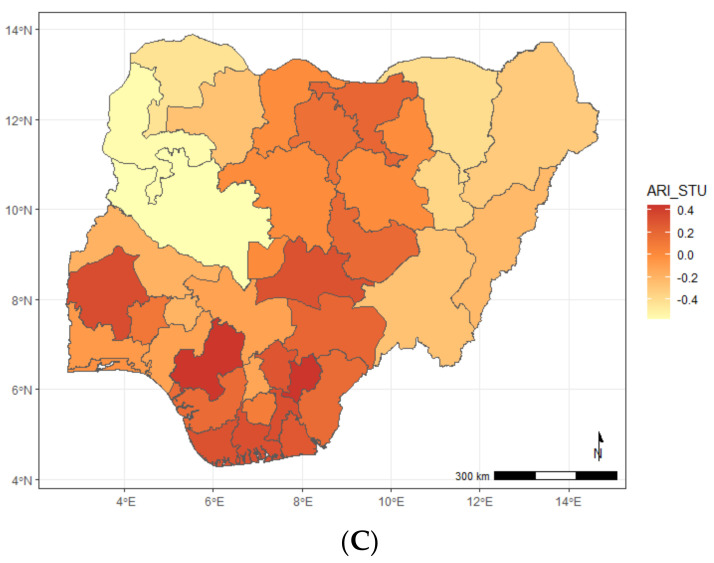
Maps showing posterior estimates of the ARI and stunting spatial shared effect by year of NDHS implementation; (**A**) 2008, (**B**) 2013, (**C**) 2018.

**Figure A6:**
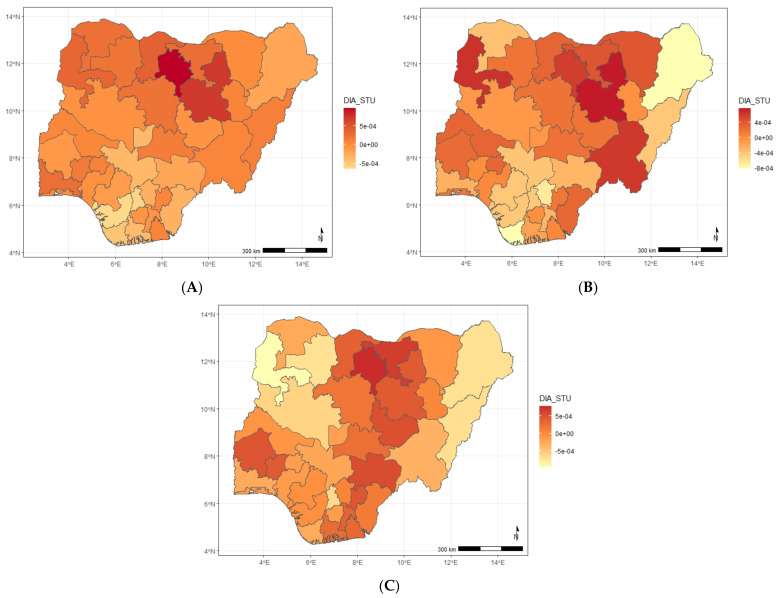
Maps showing posterior estimates of the diarrhoea and stunting spatial shared effect by year of NDHS implementation; (**A**) 2008, (**B**) 2013, (**C**) 2018.

**Figure A7:**
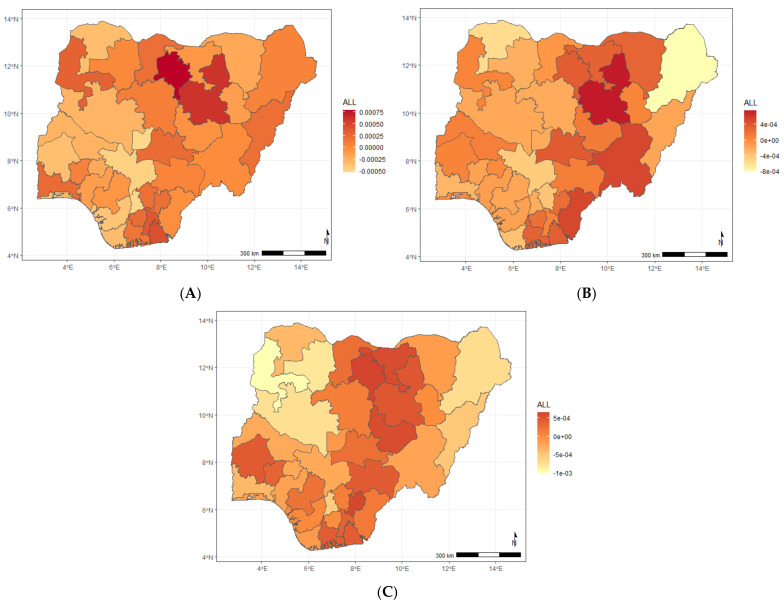
Maps showing posterior estimates of the ARI and diarrhoea and stunting spatial shared effect year of NDHS implementation; (**A**) 2008, (**B**) 2013, (**C**) 2018.

## Data Availability

The dataset used in this study are available from the DHS website https://dhsprogram.com/Data/ upon request from the MEASURE DHS program team. Written permission to use the data was obtained from Measure DHS.
